# Effect of resilience training interventions on salivary cortisol and perceived stress among Indian school students

**DOI:** 10.6026/973206300210177

**Published:** 2025-02-28

**Authors:** Jamunarani Perumalsamy, Santhi Subramaniam, Jeevithan Shanmugam, Umapathy Pasupathy

**Affiliations:** 1 Department of Psychiatric Nursing, KMCH College of Nursing, Coimbatore - 641048, Tamilnadu, India; 2 Department of Psychiatric Nursing, Sri Ramachandra Institute of Higher Education and Research (Deemed to be University) Porur, Chennai - 600116, Tamilnadu, India; 3 Department of Community Medicine, KMCH Institute of Health Sciences and Research, Coimbatore - 641014, Tamilnadu, India; 4 Department of Pediatric Medicine, Sri Ramachandra Medical College & Research Institute, Sri Ramachandra Institute of Higher Education and Research (DU), Porur, Chennai - 600116, Tamilnadu, India

**Keywords:** Adolescents, resilience training, salivary cortisol, perceived stress

## Abstract

Adolescents face significant stress, impacting their psychological and physiological health. Resilience training can enhance coping skills and reduce stress. Therefore, it is of interest to evaluate the effect of resilience training on salivary cortisol levels and perceived stress among Indian school students. Hence, a true experimental pre-test and post-test control group design was conducted with 9th-grade students in Coimbatore, India. The pilot study group underwent an 8-session resilience training intervention. Salivary cortisol and perceived stress were measured by pre - and post-intervention using standardized tools. The study group showed significant reductions in salivary cortisol (mean difference = 0.09; *p* = 0.05) and perceived stress (mean difference = 10.40; *p* = 0.001). A positive correlation (*r* = 0.28; *p* = 0.05) was observed between cortisol reduction and stress reduction. Thus, resilience training effectively reduces stress and salivary cortisol levels for adolescents.

## Background:

The ability to maintain optimal functioning in the face of stress is an essential component of well-being, especially for adolescents navigating significant developmental transitions [1]. Resilience, a protective factor against mental health challenges, plays a vital role in helping students adapt to stressors and maintain emotional balance [2]. Studies have linked higher resilience levels to reduced psychological distress and improved adjustment in academic and social contexts [3]. The prevalence of academic and mental health-related stress among school students is a growing concern globally and in India [4]. Research indicates that approximately 35-37% of Indian students report high levels of academic stress [5]. Similarly, 10-20% of adolescents worldwide experience mental health issues, with the onset often occurring between the ages of 12 and 24 years [6]. Therefore, it is of interest to evaluate the effect of resilience training on salivary cortisol levels and perceived stress among Indian school students.

## Methodology:

## Research design:

This study employed a quantitative research approach with a true experimental pre-test and post-test control group design. Two groups were formed: a study group that received resilience training interventions and a control group that did not. Randomization was applied using a lottery method to assign two schools from the SS Kulam block in Coimbatore district to the study and control groups.

## Population and sampling:

The population consisted of 9th-grade students from private and government-aided schools. A total of 20 students (10 in each group) were selected using purposive sampling. Students with moderate to high perceived stress scores were included, while those with prior resilience training or chronic illnesses were excluded.

## Intervention:

The intervention consisted of eight weekly sessions, each lasting 40 minutes, delivered during co-curricular periods. The sessions covered topics such as emotional self-awareness, anger management, coping skills, assertiveness and mindfulness. Activities included role play, video presentations and group discussions, followed by practical exercises like games and worksheets. The control group received the intervention after the post-test.

## Data collection:

Data were collected using a demographic questionnaire, salivary cortisol tests and the Perceived Stress Scale (PSS), a validated 10-item tool measuring stress levels. Pre-tests were conducted before the intervention and post-tests were conducted two weeks after to assess changes in cortisol levels and perceived stress.

## Ethical considerations:

Ethical clearance was obtained from KMCH and Sri Ramachandra Institute of Higher Education and Research. Written consent was collected from parents and students and confidentiality and anonymity were maintained.

## Statistical analysis:

Statistical analysis included descriptive statistics for demographic data, paired t-tests for within-group comparisons and independent t-tests for between-group comparisons. A correlation coefficient was used to examine the relationship between cortisol levels and stress.

## Results:

Table 1 presents the distribution of perceived stress levels before and after the intervention, highlighting that 60% of students in the study group reported low stress post-training, while the control group showed minimal changes. Table 2 compares the mean and standard deviation of salivary cortisol and perceived stress scores, demonstrating a significant reduction in cortisol (p = 0.05) and stress levels (p = 0.001) in the intervention group. Table 3 further illustrates the correlation between stress reduction and salivary cortisol reduction, revealing a significant positive relationship (r = 0.28, p = 0.05), indicating that decreased stress perception was associated with lower cortisol levels. These findings reinforce the effectiveness of resilience training in reducing both psychological and physiological markers of stress among adolescents. Figure 1 (see PDF) shows that the level of stress is reduced in post-test as compared to Pretest. These findings emphasize the effectiveness of resilience training in reducing stress among adolescents.

## Discussion:

This study explored the effectiveness of resilience training interventions in reducing salivary cortisol levels and perceived stress among Indian school students. The findings align with global evidence, highlighting the significant role of resilience-based interventions in mitigating stress and improving physiological markers of stress. The intervention group demonstrated a significant reduction in both salivary cortisol levels (mean difference = 0.09; p = 0.05) and perceived stress scores (mean difference = 10.40; p = 0.001). Notably, 60% of students in the intervention group reported low perceived stress levels post-training, compared to 0% in the control group. These findings suggest that the resilience training effectively reduced physiological and psychological stress markers, affirming its efficacy in addressing stress among adolescents. A positive correlation (r = 0.28; p = 0.05) between reductions in cortisol levels and perceived stress further underscores the interconnectedness of psychological and physiological stress responses. This finding aligns with prior research, suggesting that interventions targeting mental resilience can also influence stress biomarkers.

Conversely, the control group exhibited no significant changes in either salivary cortisol or perceived stress, reinforcing the specificity of the intervention's impact. Resilience training interventions in this study led to a significant reduction in salivary cortisol levels and perceived stress among participants. Similar results were reported by Kaligis *et al.* [7], where mental health modules for medical students showed reductions in stress perception and salivary cortisol levels over time. Studies focusing on coping strategies, such as those by Alzaharani *et al.* [8], demonstrated the efficacy of stress management programs in reducing cortisol and psychological distress among medical students. Similarly, Bottaccioli *et al.* [9] found that mindfulness-based meditation significantly decreased cortisol levels in young students, supporting the physiological impact of such interventions. This study observed a notable reduction in perceived stress among participants, consistent with findings from Iglesias *et al.* [10], where stress management programs incorporating cognitive-behavioral and relaxation techniques significantly lowered perceived stress and salivary cortisol levels. Similarly, Phang *et al.* [11] reported significant decreases in perceived stress and psychological distress among medical students undergoing mindfulness-based stress management programs. Further support comes from Hearn and Stocker [12], who demonstrated that mindfulness interventions not only reduced perceived stress but also improved assessment outcomes among medical students. While much of the existing research focuses on adults, the results of this study are particularly relevant for adolescents. Studies such as Garcia-Leon *et al.* [13] have established a strong relationship between resilience and reduced perceived stress among university students.

Dawson *et al.* [14] highlighted the efficacy of both cognitive and somatic relaxation strategies in lowering cortisol levels and stress in college students. A program by Sood *et al.* [15] for medical faculty demonstrated significant improvements in resilience, stress and overall quality of life, underscoring the importance of structured interventions. The findings of this study regarding salivary cortisol reductions align with Myint *et al.* [16], who reported similar cortisol reductions during exam periods among students exposed to resilience training. Further evidence from Adam *et al.* [17] showed diurnal cortisol variations blunted during high-stress periods, suggesting that resilience training can prevent such physiological changes. Several studies have examined the link between stress, salivary cortisol and resilience. Spiljak *et al.* (2024) [18] found that dental students with high stress had elevated cortisol, which decreased with progressive muscle relaxation. Bani-Issa *et al.* (2020) [19] reported impaired cortisol levels in female healthcare professionals, linked to poor sleep and long shifts. Borghi *et al.* (2021) observed increased cortisol during exams in pharmacy students but noted adaptive regulation over time [20]. Ng *et al.* (2004) showed that higher stress and cortisol before exams led to lower academic performance [21]. Butzer *et al.* (2016) found that school-based yoga reduced cortisol, especially in younger students. In comparison, our study confirmed that resilience training significantly lowered salivary cortisol and perceived stress in Indian school students, reinforcing the role of structured interventions in stress reduction [22].

## Limitations and future directions:

Despite the robust findings, the small sample size and short follow-up period are limitations that restrict generalizability. Future studies should include larger, diverse populations and longitudinal designs to assess the sustained impact of resilience training. Further exploration of specific intervention components, as highlighted in Iglesias *et al.* [10] and Hearn & Stocker [12], could refine program effectiveness.

## Conclusion:

The significant impact of resilience training in reducing salivary cortisol levels and perceived stress among Indian school students is highlighted in this study. Comparisons with global studies validate these findings, emphasizing the potential for structured resilience programs to enhance adolescent well-being. Incorporating such interventions into school curricula may foster resilience and reduce stress in this vulnerable population.

## Figures and Tables

**Table 1:** Distribution of pre-test and post-test levels of perceived stress scores between study and control groups

**Level of Stress**	**Pre-Test (Study Group)**	**Pre-Test (Control Group)**	**Post-Test (Study Group)**	**Post-Test (Control Group)**
Low	0 (0%)	0 (0%)	6 (60%)	0 (0%)
Moderate	7 (70%)	8 (80%)	4 (40%)	9 (90%)
High	3 (30%)	2 (20%)	0 (0%)	1 (10%)
Total	10 (100%)	10 (100%)	10 (100%)	10 (100%)

**Table 2:** Comparison of mean and standard deviation for salivary cortisol levels and perceived stress scores

**Measure**	**Group**	**Pre-Test (Mean ± SD)**	**Post-Test (Mean ± SD)**	**Mean Difference**	**t-Test Value**	**p-Value**
Salivary Cortisol	Study Group	0.29 ± 0.16	0.20 ± 0.08	0.09	2.03	0.05 (S)
	Control Group	0.29 ± 0.17	0.28 ± 0.15	0.01	1.38	0.20 (NS)
Perceived Stress Score	Study Group	24.90 ± 4.04	14.50 ± 4.45	10.4	5.53	0.001 (S)
	Control Group	24.50 ± 4.38	23.00 ± 4.88	1.5	1.38	0.20 (NS)
Note: S = Significant;
NS = Not Significant

**Table 3:** Correlation between stress reduction and salivary cortisol reduction scores

**Group**	**Stress Reduction Score (Mean ± SD)**	**Salivary Cortisol Reduction Score (Mean ± SD)**	**Correlation Coefficient (r)**	**p-Value**	**Interpretation**
Study Group	10.40 ± 5.95	0.09 ± 0.12	0.28	0.05 (S)	Significant positive fair correlation
Control Group	1.50 ± 3.44	0.01 ± 0.03	0.12	0.38 (NS)	Positive poor correlation
